# Complete chloroplast genome and comparison of herbicides toxicity on *Aeschynomene indica* (Leguminosae) in upland direct-seeding paddy field

**DOI:** 10.1186/s12864-024-10102-x

**Published:** 2024-03-14

**Authors:** Yuan Gao, TianYu Chen, Jiaqi Long, Guohui Shen, Zhihui Tian

**Affiliations:** 1 Eco-Environmental Protection Research Institute, Shanghai Academy of Agricultural Sciences, 201403 Shanghai, China; 2 School of Chemical and Environmental Engineering, Shanghai Institute of Technology, 201418 Shanghai, China

**Keywords:** Indian jointvetch, Chloroplast genome, Related species, Upland direct-seeding rice, Herbicide

## Abstract

**Background:**

Indian jointvetch (*Aeschynomene indica*) is a common and pernicious weed found in the upland direct-seeding rice fields in the lower reaches of the Yangtze River in China. However, there are few reports on the degree of harm, genetic characteristics, and management methods of this weed. The purpose of this study is to clarify the harm of Indian jointvetch to upland direct-seeding rice, analyze the genetic characteristics of this weed based on chloroplast genomics and identify its related species, and screen herbicides that are effective in managing this weed in upland direct-seeding rice fields.

**Results:**

In a field investigation in upland direct-seeding rice paddies in Shanghai and Jiangsu, we determined that the plant height and maximum lateral distance of Indian jointvetch reached approximately 134.2 cm and 57.9 cm, respectively. With Indian jointvetch present at a density of 4/m^2^ and 8/m^2^, the yield of rice decreased by approximately 50% and 70%, respectively. We further obtained the first assembly of the complete chloroplast (cp.) genome sequence of Indian jointvetch (163,613 bp). There were 161 simple sequence repeats, 166 long repeats, and 83 protein-encoding genes. The phylogenetic tree and inverted repeat region expansion and contraction analysis based on cp. genomes demonstrated that species with closer affinity to *A. indica* included *Glycine soja*, *Glycine max*, and *Sesbania cannabina*. Moreover, a total of 3281, 3840, and 3838 single nucleotide polymorphisms were detected in the coding sequence regions of the cp. genomes of *S. cannabina* voucher IBSC, *G. soja*, and *G. max* compared with the *A. indica* sequence, respectively. A greenhouse pot experiment indicated that two pre-emergence herbicides, saflufenacil and oxyfluorfen, and two post-emergence herbicides, florpyrauxifen-benzyl and penoxsulam, can more effectively manage Indian jointvetch than other common herbicides in paddy fields. The combination of these two types of herbicides is recommended for managing Indian jointvetch throughout the entire growth period of upland direct-seeding rice.

**Conclusions:**

This study provides molecular resources for future research focusing on the identification of the infrageneric taxa, phylogenetic resolution, and biodiversity of Leguminosae plants, along with recommendations for reliable management methods to control Indian jointvetch.

**Supplementary Information:**

The online version contains supplementary material available at 10.1186/s12864-024-10102-x.

## Background

The genus *Aeschynomene* in the family Leguminosae is widely distributed in tropical and subtropical regions of the world, mostly in America, Africa, and Asia, with approximately 200 species identified to date [1–3]. *Aeschynomene* is an annual herbaceous plant that grows as shrubs, with shield-shaped stipules, butterfly-shaped flowers, and segmented leaves connected by diaphragms [4]. Only one species of this genus has been found in China to date, namely *A. indica* (Indian jointvetch) [2]. It usually germinates in April and its seeds mature in October. The germination of Indian jointvetch seeds mainly depends on the temperature during the day [5]. Research has shown that the seeds of Indian jointvetch have dormancy characteristics, which can survive for about six months in the soil seed bank and mechanical scarification is an effective method to break dormancy [6].

Although this plant does not germinate under flooded conditions, once it grows, Indian jointvetch indirectly affects crop productivity by competing for resources, hindering harvesting operations, and reducing grain and seed quality [7–9]. Moreover, it has been confirmed that mixing seeds of *A. indica* into rice as pig feed can induce the development of pig diseases [1]. *A. indica* was reported to be a weed in irrigated rice fields previously [10, 11] and was considered the third most troublesome weed after weedy rice and barnyard grass in certain rice-producing areas [12]. However, *A. indica* was recently found to impair the growth of upland direct-seeding rice in the lower reaches of the Yangtze River in China (Additional File 3, Figure S1). Because the planting area of upland direct-seeding rice is increasing owing to the global shortage of labor and water resources, more and more attention has been paid to the serious weed problem [13–16]. Distinguishing plants of the genus *Aeschynomene* is very difficult, and managing these weeds in rice fields is also very challenging presently [3]. Therefore, further research is required to elucidate the characteristics of this species, including the genetic characteristics, degree of harm induced, and responses to available herbicides for effective management and control.

Chloroplasts are the main organelles involved in photosynthesis in photosynthetic plants or algae [17]. Chloroplasts have their own distinct genetic material with characteristics of non-recombination, haploid, and single-parent inheritance, which leads to a highly conserved genome; therefore, analysis of the chloroplast (cp.) genome can provide very rich evolutionary information [18–20]. Additionally, the cp. genome is small and easy to obtain, offering unique research value in phylogeny, species identification, and population genetics [21]. The cp. genome has a typical tetrad structure, with large single copy (LSC) and small single copy (SSC) regions separated by inverted repeats (IRs) [22–24]. Previous studies have shown that in the context of network evolution (i.e., hybridization) and polyploidy, analysis of the cp. genome is particularly useful for characterizing the phylogenetic and historical aspects of most plant lineages [25–27]. However, information on the composition, structure, interspecific differences, and evolutionary relationships of *A. indica* based on its cp. genome is limited. Therefore, exploring the cp. genomic information of *A. indica* can provide a theoretical basis for the management or utilization of this species and predict the possibility of evolution of related species into paddy weeds.

Current weed control methods in upland direct-seeding rice fields mainly include cultural weed management practices and physical methods such as deep plowing, germination, mechanical weeding, and hand-pulling weeding, along with chemical methods using herbicides [28–30]. Chemical methods for weed control in upland direct-seeding rice fields have specific advantages of obtaining higher yields with relatively lower labor costs [30–32]. *Aeschynomene* weeds are often controlled using a combination of imazapyr and imazapic herbicides [3]. However, this genus of weed has been found to exhibit imazapic resistance [33]. In addition, this herbicide is typically considered to be unsafe for rice growth. To our knowledge, there have been no systematic studies on the toxicity of common herbicides used for *A. indica* in rice fields. Therefore, screening for suitable herbicides to effectively control *A. indica* is of great significance to achieve a high and optimal yield of upland direct-seeding rice.

To address these questions, we performed an in-depth analysis of the genetic characteristics and herbicide response of *A. indica* growing in the upland direct-seeding rice fields of Shanghai, China. We studied the morphological characteristics of this weed in rice fields and its effect on rice yield. The cp. genome of *A. indica* was then sequenced and compared to those of other species to analyze their genetic characteristics and evolutionary relationships. Finally, the toxicities of common herbicides currently used in rice fields in China against *A. indica* were evaluated in a greenhouse pot experiment to determine a reasonable chemical control method. Our study thus provides a theoretical and practical foundation for gaining a better understanding of the damage of the troublesome dicotyledonous weed *A. indica* imposes in upland direct-seeding rice fields and facilitating strategies for its effective management.

## Methods

### Plant materials

Indian jointvetch seeds were collected from upland direct-seeding rice fields in Jinshan District, Shanghai, China (N 30.81°, E 121.18°) in October 2021. The seeds were dried to constant weight after collection and stored at 4 °C until planted.

### Determination of field traits

Plant height and the maximum lateral distance of Indian jointvetch growing in upland direct-seeding rice fields in Shanghai and Jiangsu Province were measured in October 2021. More than 20 plants were randomly selected; however, no more than three plants were sampled from each paddy field. Seeds were collected, dried to a constant weight, and weighed. A total of 294 upland direct-seeding rice fields were investigated, fields with Indian jointvetch were recorded, and their frequency was calculated.

Subsequently, 0, 1, 2, 3, and 4 Indian jointvetch seedlings grown in a greenhouse previously were transplanted into a 1 m^2^ upland direct-seeding rice planting area and all other weeds were manually removed. When the upland direct-seeding rice had matured, the effective spike number, grains per spike, and 1000-seed weight of upland direct-seeding rice seeds were measured. Each treatment contains three biological replicates and the entire experiment was repeated twice. Data of percentages of fresh weight compared to the control (none transplanted Indian jointvetch) were subjected to ANOVA. For comparison of the differences in the rice yield indicators among the five groups, the Duncan’s Multiple Range Test (*P* < *0.05*) was used. ANOVA was conducted using SPSS version 20 (SPSS, Chicago, IL, USA).

### Construction of the *A. indica* cp. genome

#### DNA sequencing and genome assembly

Total genomic DNA from ten Indian jointvetch at the seedling stage was extracted using a modified cetyltrimethylammonium bromide method and applied to construct a 500-bp paired-end library using the NEBNext Ultra DNA Library Prep Kit (New England Biolabs, Ipswich, MA, USA) for next-generation sequencing on the Illumina NovaSeq 6000 platform (Berry Genomics Co., Ltd., Beijing, China). Approximately 2.35 Gb of raw data from Indian jointvetch were generated with 150-bp paired-end read lengths. *De novo* assembly was performed using NOVOPlasty v4.2 software (https://github.com/ndierckx/NOVOPlasty, accessed March 24, 2023). Detailed sequencing and assembly methods are described in our previous study [34].

#### Genome component analysis and gene annotation

Genes encoding proteins, tRNAs, and rRNAs in the cp. genome of Indian jointvetch were predicted using GeSeq software (https://chlorobox. mpimp-golm. mpg. de/geseq. com ml/, accessed March 24, 2023). The protein sequences of cp. genes were compared with known proteins in databases using BLASTP (https://ncbiinsights.ncbi.nlm.nih.gov/tag/blastp/, accessed March 24, 2023) (e-value < 1 × 10^− 5^). Because there may be more than one alignment result for each sequence, only one optimal alignment result was reserved for database alignment for the given gene to ensure its biological significance. The amino acid sequences of Indian jointvetch were aligned with sequences included in the Non-Redundant Protein Sequence (http://www.ncbi.nlm.nih.gov/, accessed March 24, 2023), Swiss-Prot (http://www.ebi.ac.uk/uniprot, accessed March 24, 2023), Clusters of Orthologous Groups, Kyoto Encyclopedia of Genes and Genomes (KEGG; http://www.genome.jp/kegg/, accessed March 24, 2023), and Gene Ontology (GO; http://geneontology.org/, accessed March 24, 2023) databases to obtain functional annotation information for the coding genes.

### Analysis of genetic relationships and identification characteristics

#### Phylogenetic analysis

Nineteen cp. genomes of common plants, including the model plant of dicotyledons *Arabidopsis thaliana*, common plants growing in rice fields (*Oryza sativa* Indica, *O. sativa* Tropical Japonica, *Echinochloa oryzoides*, *Eclipta prostrata*, *Ammannia arenaria*, *Ammannia multiflora*, *Cyperus iria*, *Cyperus difformis*), and common Leguminosae plants (*Vicia sepium*, *Sesbania cannabina*, *Medicago sativa*, *Medicago truncatula*, *Medicago polymorpha*, *Glycine max*, *Glycine soja*, *Astragalus sinicus*), were downloaded from the National Center for Biotechnology Information (NCBI) database (see Additional File 1, Table S1 for accession numbers) and subject to phylogenetic analysis to determine their evolutionary relationships with Indian jointvetch. The sequences were aligned using ClustalW (v2.0.12) (http://www.clustal.org/clustal2/, accessed April 4, 2023) with default settings. The DNA substitution model was utilized based on the Akaike information criterion [35]. The phylogenetic tree was constructed by the maximum-likelihood (ML) method using PhyML v3.0 (htp://ww.atgc-montpeller. fr/phyml/, accessed April 4, 2023) and bootstrap values were calculated for 1000 replicates [36, 37]. The tree building model was finally evaluated using jModelTest 2.1.10 (https://github.com/ddarriba/jmodeltest2, accessed April 4, 2023), with the best model “GTR + I + G.”

#### Contraction and expansion analysis of IR regions

We performed IR contraction and expansion analysis for the cp. genomes of Indian jointvetch and species with closer genetic relationships selected based on the phylogenetic analysis. The four quadripartite structures (LSC, SSC, and two IR repeat regions) of each cp. genome were compared, and changes in the copy number of related genes caused by contraction and expansion of the IR or pseudogenes, resulting in boundary regions, were analyzed. Genes that crossed or were adjacent to these boundaries were identified. In addition, the length and distance from the boundaries of these genes were analyzed.

#### Single nucleotide polymorphism (SNP) analysis

Using MUMmer software (http://mummer.sourceforge.net/, accessed April 4, 2023), the cp. genome sequences of Indian jointvetch (as the reference) and closely related species, based on phylogenetic and contraction/expansion IR analyses, were completely aligned, and preliminary filtering was performed to detect potential SNP sites. Sequences of 100 bp on both sides of the candidate SNP site of the reference sequence were extracted and aligned with the assembly results using BLAT v35 software (http://hgdownload.soe.ucsc.edu/admin/exe/linux.x86_64/blat/, accessed April 4, 2023) to verify the SNP site. If the alignment length was less than 101 bp, it was considered an unreliable SNP and was removed; if the alignment was repeated multiple times, the SNP was considered a repetitive region and was removed, resulting in only reliable SNPs.

### Herbicide sensitivity assays

We determined the susceptibility of Indian jointvetch to common herbicides applied in rice fields using a pot experiment in the greenhouse. The soil was a middle loam obtained from a farmland in the suburbs of Shanghai, China, where herbicides had not been used. Five seeds were sown in each plastic cup (7 × 7 × 7 cm), water was added until saturation, a fine layer of soil was added, and the seeds were treated with herbicides (i.e., pre-emergence treatment) using a 3WP-2000 walking-type spray system (Nanjing Agricultural Mechanization Research Institute of the Ministry of Agriculture, China). Each treatment was performed with 30 mL of liquid (450 L ha^− 1^ water) using a fan-shaped nozzle. Control plants were sprayed with the same amount of water as the herbicide for the treated plants. The seedlings were then placed in a greenhouse for cultivation. When the seeds of the control group germinated and the treatment group showed a significant gradient, the Indian jointvetch plants were cut and weighed. Five seeds were sown and cultivated at the three-leaf stage for post-emergence herbicide application using the same method. After 21 days, the above-ground Indian jointvetch in each cup was cut and weighed. The dose and details of the herbicides are listed in Table 1. The experiment included three to four biological replicates and the entire experiment was repeated twice. The effective rate of each herbicide causing 50% reduction in plant growth (GR_50_) was determined using the four-parameter logistic function with the “drc” add-on package [38] in the R 3.1.3 Language and Environment for Statistical Computing [39]. This model is defined as follows:

$$ \text{Y}=\text{c}+\left\{\right(\text{d}-\text{c})/(1+\text{e}\text{x}\text{p}\left(\text{b}\right(\text{l}\text{o}\text{g} \text{x}-\text{l}\text{o}\text{g} \text{e}\left)\right)\left)\right\}$$

where parameter *e* represents GR_50_ as the dose producing a response halfway between the upper limit *d* and the lower limit *c*, and parameter *b* denotes the relative slope around *e*.

**Table 1:** Information of the herbicides used in this study

Type	Herbicides	Manufacturer	Dose (g a.i. ha^− 1^)
Pre-emergence	Bensulfuron-methyl	Zhejiang Tianyi Biotechnology Co.,Ltd., Shaoxing, Zhejiang, China	0, 5.625, 11.25, 22.5, 45, 90, 180
	Butralin	Shield Corporation, Fuzhou, Jiangxi, China	0, 135,270, 540, 1080, 2160, 4320
	Oxyfluorofen	Yifan Biotechnology Group Co., Ltd., Wenzhou, Zhejiang, China	0, 11.25, 22.5, 45, 90, 180, 360
	Saflufenacil	BASF SE, Ludwigshafen, Rheinland-Pfalz, Germany	0, 0.984375, 1.96875, 3.9375, 7.875, 15.75, 31.5
Post-emergence	Pyrazosulfuron-ethyl	Liben Crop Technology Co., Ltd., Liangyungang, Jiangsu, China	0, 0.9375, 1.875, 3.75, 7.5, 15, 30
	2-methyl-4-chlorophenoxyacetic acid	Jiangsu Jian Gu Chemical Industry Co., Ltd., Suqian, Jiangsu Province, China	0, 24.375, 48.75, 97.5, 195, 390, 780
	Penoxsulam	Corteva Agriscience, Wilmington, DE, USA	0, 0.9375, 1.875, 3.75, 7.5, 15, 30
	Quinclorac	Jiangsu Repont Agrochemical Co., Ltd., Changzhou, Jiangsu, China	0, 18.75, 37.5, 75, 150, 300, 600
	Florpyrauxifen-benzyl	Corteva Agriscience, Wilmington, DE, USA	0, 0.28125, 0.5625, 1.125, 2.25, 4.5, 9
	Pyraquinate	Shandong Cynda (Chemical) Co., Ltd., Jinan, Shandong, China	0, 4.6875, 9.375, 18.75, 37.5, 75, 150

## Results

### Impact of *A. indica* on rice growth

**Fig. 1:**
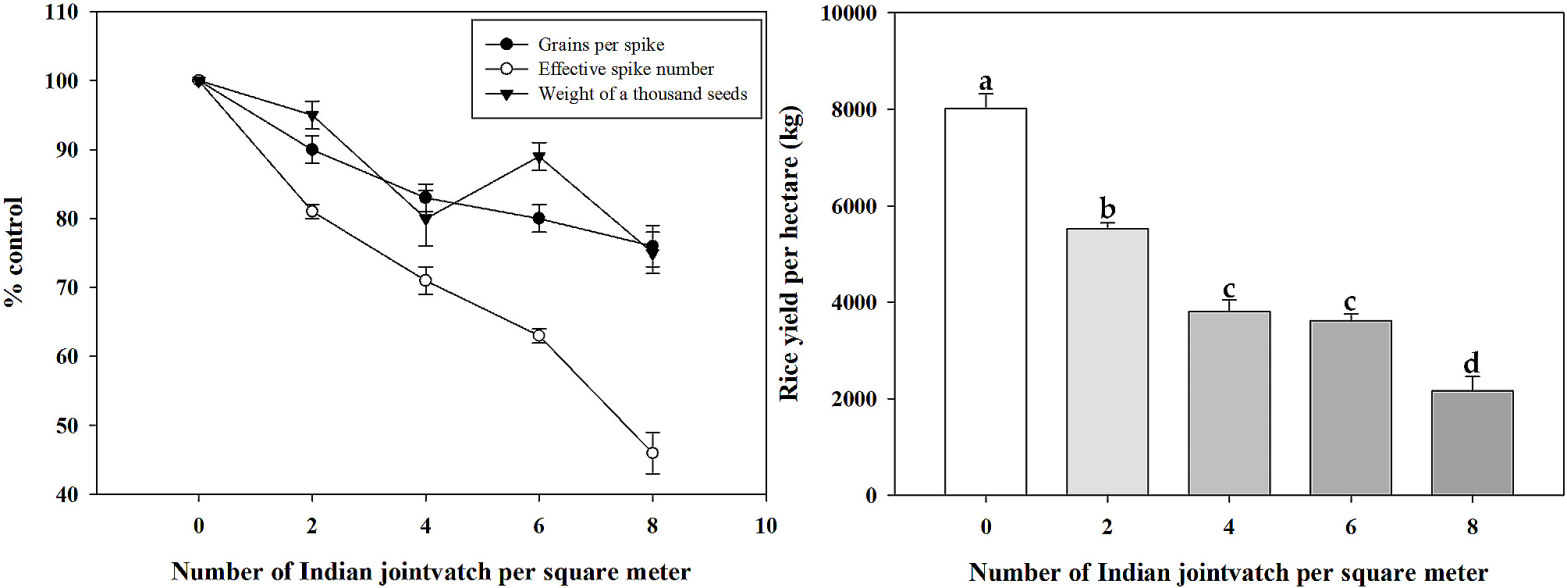
Effect of the number of Indian jointvetch plants on rice yield in the field. The standard errors of the means are described by vertical bars. ANOVA significance groupings are shown as **a**, **b**, **c**, and **d** (*P* < 0.05)

We first investigated the morphological features of Indian jointvetch during seed maturity in upland paddy fields in the lower reaches of the Yangtze River in China. The average height of Indian jointvetch was 134.2 ± 3.7 cm, which was higher than that of the rice (approximately 90 cm). The mean maximum lateral distance was 57.9 ± 2.1 cm. The seeds presented a kidney shape (Additional File 3, Figure S2) with a 1000-seed weight of 10.39 ± 0.055 g. The frequency of occurrence of this weed in upland paddy fields in our survey was 13.85 ± 2.22%. From the perspective of rice damage, as the number of Indian jointvetch plants increased, the effective spike number and grains per spike of upland direct-seeding rice exhibited a significant downward trend. Rice seed weights also showed an overall downward trend with an increasing number of Indian jointvetch plants. Specifically, when the number of Indian jointvetch was 0, 2, 4, 6, and 8, the yields of upland direct-seeding rice were 8019.3, 5534.4, 3783.2, 3611.4, and 2092.5 kg/ha, respectively (Fig. 1).

### Characteristics of the *A. indica* cp genome

#### Cp genome map

**Fig. 2:**
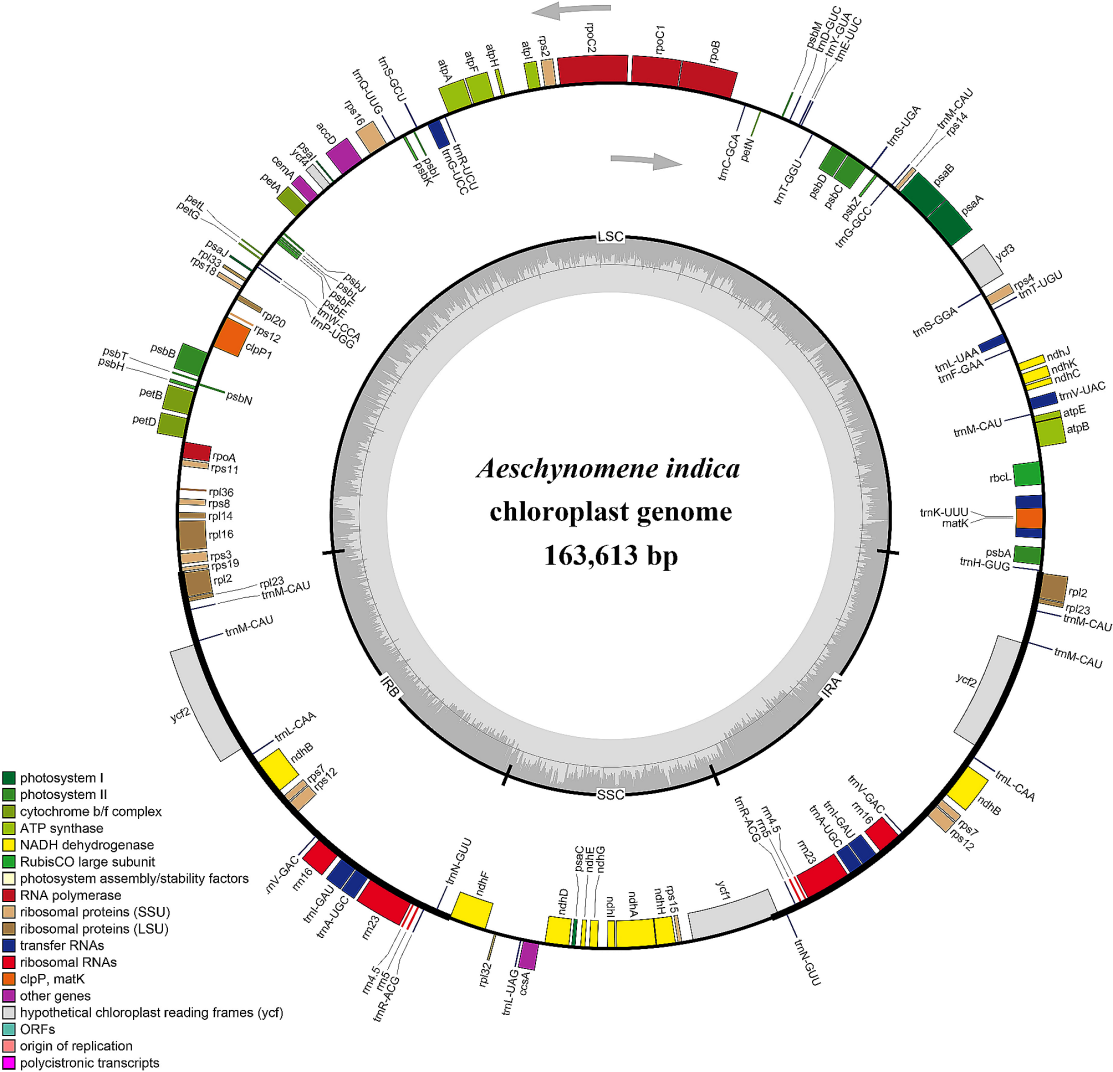
Assembly, size, and features of the chloroplast genome of Indian jointvetch. The genes outside the circle are transcribed in the counterclockwise direction and the genes inside the circle are transcribed in the clockwise direction. Different colors of genes represent different functions. The dark gray area and light gray area of the inner circle represent the GC and AT content of the genome, respectively

After trimming low-quality fragments from the raw data, 33,624,994 clean reads with a GC content of 35.93% were mapped to the complete cp. genome of Indian jointvetch. *De novo* assembly resulted in a circular genome of 163,613 bp in length (Fig. 2). The raw reads were deposited in the NCBI GenBank database (accession number: PRJNA963187). The complete cp. genome displayed the typical quadripartite structure of most angiosperms, including an LSC region, SSC region, and pair of IRs (IRa and IRb). The lengths of the LSC, SSC, and IR regions were 88,562, 19,801, and 27,625 bp, respectively, and the intergenic region length was 86,150 bp. The Indian jointvetch cp. genome contains 83 protein-coding genes (Table 2).

**Table 2:** Summary of chloroplast genome features in Indian jointvetch

Genome Features	Indian jointvetch
Genome size (bp)	163,613
LSC length (bp)	88,562
SSC length (bp)	19,801
IR length (bp)	27,625
Intergenic region Length (bp)	86,150
Overall GC content (%)	35.54
GC content of LSC (%)	32.77
GC content of SSC (%)	28.71
GC content of IR (%)	42.41
Number of Protein-coding genes	83

#### Cp genome components

The cp. genome of Indian jointvetch contained 39 tRNA genes and eight rRNA genes (Table 3). There were 70 protein-coding and 26 tRNA genes located within the LSC; 10 protein-coding genes, 10 tRNA-coding genes, and four rRNA-coding genes located within IRb or IRa; and 13 protein-coding genes and one tRNA gene located within the SSC region (Fig. 2). All 83 genes encoding proteins in the cp. genome of Indian jointvetch were functionally annotated, which mainly belonged to the photosynthesis and self-replication categories. The gene names, groups, and categories are listed in Table 4. The genes were mainly associated with GO biological processes (Additional File 3, Figure S3), and were associated with KEGG energy production and conversion, translocation, ribosomal structure, and biogenesis pathways (Additional File 3, Figure S4). In total, 161 simple sequence repeats (SSRs) were identified in the Indian jointvetch cp. genome. There were eight SSRs on IRa or IRb, 114 on the LSC region, and 31 on the SSC region. In total, 166 long repeats (LRs) were identified in the cp. genome of Indian jointvetch (Table 5).

**Table 3:** Non-coding RNAs in the Indian jointvetch chloroplast genome

Type	ncRNA number	Total length (bp)	Average length (bp)	Length/ Genome (%)
tRNA	39	**2943**	75	1.8
rrn23	2	**5630**	**2815**	**3.44**
rrn4.5	2	208	104	0.13
rrn16	2	2982	1491	1.82
rrn5	2	242	121	0.15

**Table 4:** Annotated genes of the Indian jointvetch chloroplast genome

Category	Groups	Genes	
Photosynthesis	Subunits_of_photosystem_I	*psaA*, *psaB*, *psaC*, *psaI*, *psaJ*	
	Subunits_of_photosystem_II	*psbA*, *psbB*, *psbC*, *psbD*, *psbE*, *psbF*, *psbH*, *psbI*, *psbJ*, *psbK*, *psbL*, *psbM*, *psbN*, *psbT*, *psbZ*	
	Subunits_of_NADH_dehydrogenase	*ndhA*, *ndhB*, *ndhB*, *ndhC*, *ndhD*, *ndhE*, *ndhF*, *ndhG*, *ndhH*, *ndhI*, *ndhJ*, *ndhK*	
	Subunits_of_cytochrome_b/f_complex	*petA*, *petB*, *petD*, *petG*, *petL*, *petN*	
	Subunits_of_ATP_synthase	*atpA*, *atpB*, *atpE*, *atpF*, *atpH*, *atpI*	
	Large_subunit_of_Rubisco	*rbcL*	
Self-replication	Large_subunits_of_ribosome	*rpl14*, *rpl16*, *rpl2*, *rpl2*, *rpl20*, *rpl23*, *rpl23*, *rpl32*, *rpl33*, *rpl36*	
	Small_subunits_of_ribosome	*rps11*, *rps12*, *rps12*, *rps14*, *rps15*, *rps16*, *rps18*, *rps19*, *rps19*, *rps2*, *rps3*, *rps4*, *rps7*, *rps7*, *rps8*	
	DNA-dependent_RNA_polymerase	*rpoA*, *rpoB*, *rpoC1*, *rpoC2*	
	Ribosomal_RNAs	*8 rRNA*	
	Transfer_RNAs	*39 tRNAs*	
Other genes	Maturase	*matK*	
	Protease	*clpP1*	
	Envelope_membrane_protein	*cemA*	
	Acetyl-CoA_carboxylase		*accD*
	C-type_cytochrome_synthesis_gene	*ccsA*	
	Translation_initiation_factor		
	protochlorophillide_reductase_subunit		
Genes unknown	Proteins_of_unknown_function	*Ycf1*, *ycf2*, *ycf3*, *ycf4*	

**Table 5:** Single sequence repeats (SSRs) and long repeats (LRs) in the Indian jointvetch chloroplast genome

Type of repeats	Distribution	Number
**SSR**	Genome	161
	Coding	22
	IRa/b	8
	LSC	114
	SSC	31
**LR**	Hamming Distance = 0	22
	Hamming Distance = 1	6
	Hamming Distance = 2	28
	Hamming Distance = 3	110

### Genetic similarity analysis

#### Phylogenetic analysis

**Fig. 3:**
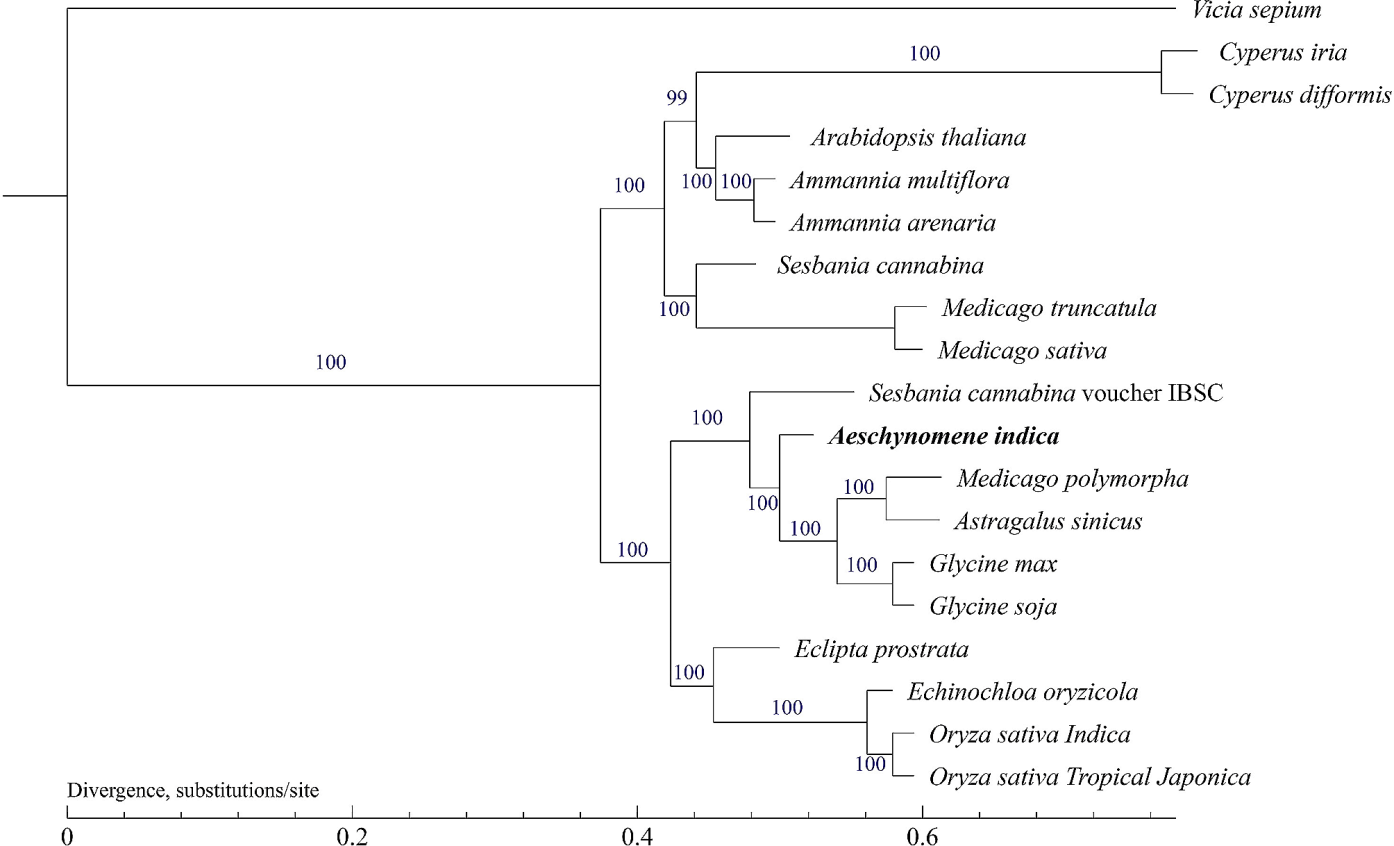
Phylogenetic tree constructed using the maximum-likelihood method based on alignments of complete chloroplast genome sequences. The numbers at the nodes indicate bootstrap values from 1000 replicates. If the bootstrap value is 100, this number is not shown on the nodes. The species newly sequenced in this study is shown in bold

Bayesian inference of 19 complete cp. genomes showing the same topology demonstrated that Indian jointvetch clustered into a single clade (Fig. 3). Indian jointvetch, *A. sinicus*, *M. polymorpha*, *G. soja*, and *G. max* have the most recent common ancestor (MRCA) (BS = 100 for the ML tree), and this group has an MRCA with *S. cannabina* voucher IBSC voucher (BS = 100 for the ML tree). The closest relatives to these plants were *E. prostrata*, *O. sativa* Indica, *O. sativa* Tropical Japonica, and *E. oryzicola* (BS = 100 for the ML tree). In addition, the group formed by *S. cannabina-*1, *M. sativa*, *M. polymorpha*, *A. thaliana*, *C. iria*, *C. difformis*, *A. arenaria*, and *A. multiflora* was closely related to the Indian jointvetch group. The most distantly related plant to Indian jointvetch was *V. sepium*.

#### IR expansion and contraction

**Fig. 4:**
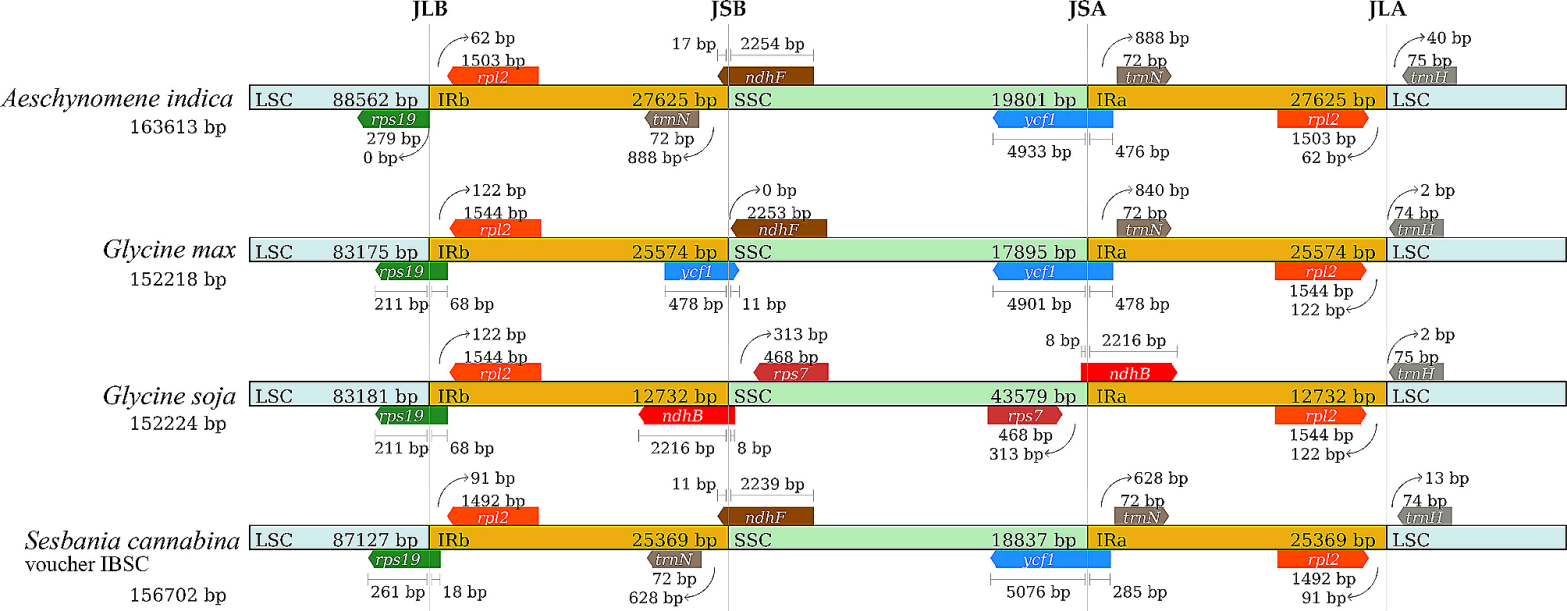
Comparison of large sequence copy (LSC), inverted repeat (IRb, IRa), and small sequence copy (SSC) border regions of the chloroplast genomes of Indian jointvetch and related species

To further observe the potential genetic relationships among the most closely related species to Indian jointvetch, namely *S. cannabina* voucher IBSC, *G. soja*, and *G. max*, based on the contraction and expansion of IR regions, the gene variations at the IR/SSC and IR/LSC boundary regions of the four species were compared (Fig. 4); *A. sinicus* and *M. polymorpha* do not have complete IR regions and were therefore excluded from this analysis. The genes *rps19*/ *rpl2*, *trnN*/ *ndhF*, *ycf1*/ *trnN*, and *rpl2*/ *trnH* were identified at the junctions of the LSC/IRb, IRb/SSC, SSC/IRa, and IRa/LSC regions, respectively, in Indian jointvetch. Among them, *ndhF* crosses IRb/SSC and *ycf1* crosses SSC/IRa. Among all tested species, the closest to Indian jointvetch was *S. cannabina* voucher IBSC with respect to the expansion and contraction of IR regions; these two species exhibited identical boundary genes with the only difference being the length of the genes or the distance between the genes and the boundary.

#### SNP analysis

SNP analysis was performed to compare the differentiation among Indian jointvetch, *S. cannabina* voucher IBSC, *G. soja*, and *G. max*. A total of 4659 SNPs were detected in *S. cannabina* voucher IBSC, including 1378 SNPs in the intergenic spacer regions and 3281 SNPs in the coding sequence regions (Table 6; Additional File 2, Table S2). The nonsynonymous to synonymous substitution ratio was 0.626. Two SNPs were detected at the start codon and eight SNPs were detected at the stop codon. The numbers of SNPs in the cp. genomes of *G. soja* and *G. max* were much higher than that for *S. cannabina* voucher IBSC compared to the Indian jointvetch cp. genome.

**Table 6:** Single nucleotide polymorphisms (SNPs) in *S. cannabina* voucher IBSC, *G. soja*, and *G. max* chloroplast genomes compared to the Indian jointvetch chloroplast genome

Species	Start	Stop	Synonymous	Nonsynonymous	CDS	Intergenic	Total SNPs
*S. cannabina* voucher IBSC	2	8	2000	1252	3281	1378	4659
*G. soja*	1	7	2344	1469	3840	1595	5435
*G. max*	1	7	2342	1470	3838	1594	5432

CDS, coding sequence

### Chemical herbicide effects

#### Toxicity of pre-emergence herbicides

**Fig. 5:**
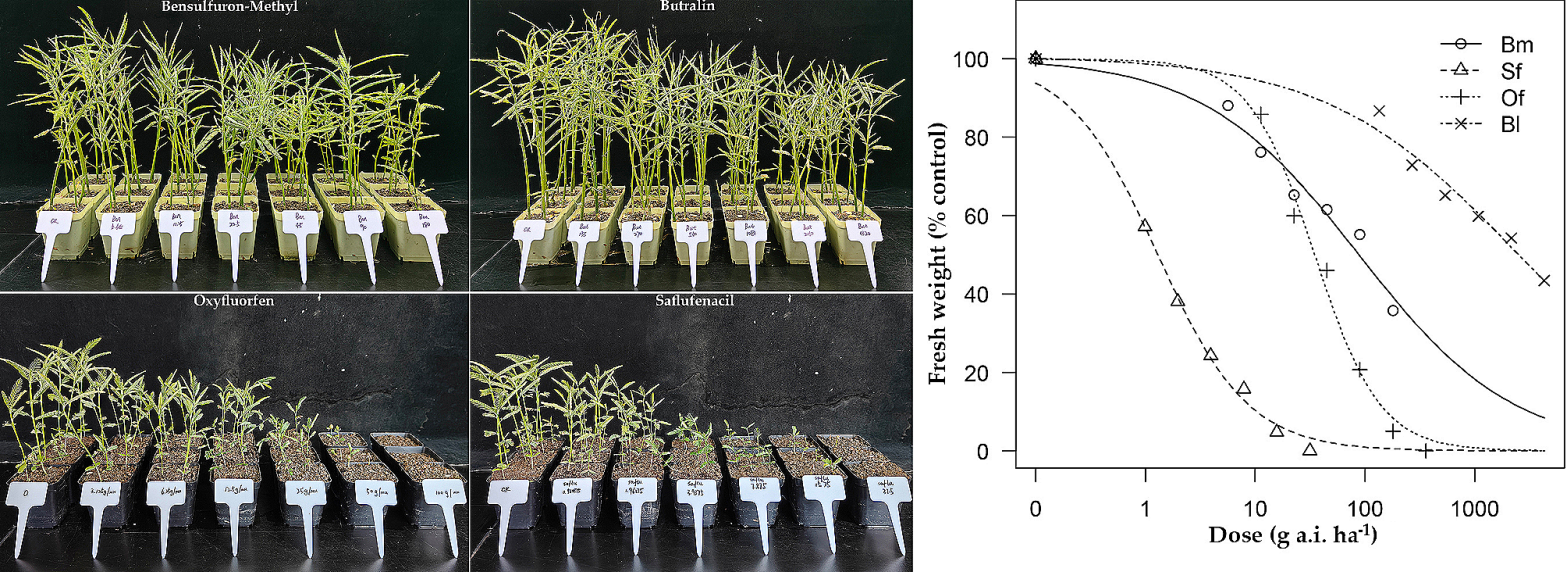
Dose–response analyses for the response of Indian jointvetch to pre-emergence herbicides. The *X*-axis represents the dose (g a.i. ha^− 1^) and the *Y*-axis represents the percentage of fresh weight to that of the untreated control. “Bm,” “Sf,” “Of,” and “Bl” represent the herbicides bensulfuron-methyl, saflufenacil, oxyfluorofen, and butralin, respectively

In pre-emergence application, as the doses of oxyfluorofen and saflufenacil increased, Indian jointvetch was significantly inhibited on the 14th day after herbicide treatment and showed a gradient decrease pattern thereafter. However, upon treatment with the other two herbicides, bensulfuron-methyl and butralin, Indian jointvetch did not show significant growth inhibition until 21 days after treatment (Fig. 5). The toxicity calculation showed that the GR_50_ value of oxyfluorofen on Indian jointvetch was 34.9 g a.i. ha^− 1^ and the GR_90_ value was 157.5 g a.i. ha^− 1^; the GR_50_ value of saflufenacil on Indian jointvetch was 1.3 g a.i. ha^− 1^ and the GR_90_ value was 10.3 g a.i. ha^− 1^ (Fig. 5).

#### Toxicity of post-emergence herbicides

**Fig. 6:**
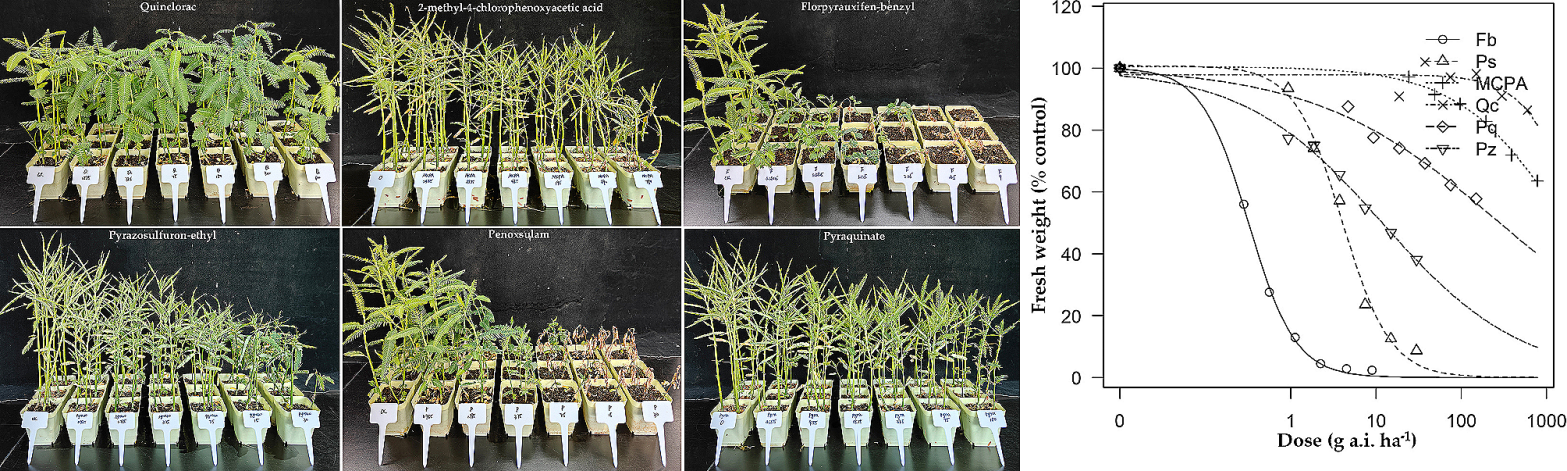
Dose–response analyses for response of Indian jointvetch to post-emergence herbicides. The *X*-axis represents the dose (g a.i. ha^− 1^) and the *Y*-axis represents the percentage of fresh weight to that of the untreated control. “Fb,” “Ps,” “MCPA,” “Qc,” “Pq,” and “Pz” represent the herbicides florpyrauxifen-benzyl, penoxsulam, 2-methyl-4-chlorophenoxyacetic acid, quinclorac, pyraquinate, and pyrazosulfuron-ethyl, respectively

With post-emergence application, with the increase in the doses of florpyrauxifen-benzyl, pyrazosulfuron-ethyl, and penoxsulam, Indian jointvetch was significantly inhibited on the 21st day after herbicide treatment and showed a decreasing gradient thereafter; the other three herbicides, quinclorac, 2-methyl-4-chlorophenoxyacetic acid, and pyraquinate, did not have a significant inhibitory effect on the growth of Indian jointvetch. From the toxicity calculation, the GR_50_ value of florpyrauxifen-benzyl on Indian jointvetch was 0.3 g a.i. ha^− 1^ and the GR_90_ value was 1.3 g a.i. ha^− 1^. The GR_50_ value of penoxsulam on Indian jointvetch was 4.0 g a.i. ha^− 1^ and the GR_90_ value was 16.8 g a.i. ha^− 1^. The GR_50_ value of pyrazosulfuron-ethyl on Indian jointvetch was 11.9 g a.i. ha^− 1^ and the GR_90_ value was g a.i. ha^− 1^ 737.5, but this far exceeds its safe usage limit (45.0 g a.i. ha^− 1^) (Fig. 6).

## Discussion

According to the results of our on-site investigation, Indian jointvetch causes serious harm to upland direct-seeding rice fields in the lower reaches of the Yangtze River in China. Upland direct-seeding rice is an important food crop grown in areas with limited water resources presently. However, rampant weeds are an obstacle to the cultivation of upland direct-seeding rice [14–16]. The rough management practice of the upland direct-seeding rice field system has further aggravated weed ravages. Although studies have shown that Indian jointvetch can be considered a green fertilizer that is beneficial for rice growth [40], we obtained contrasting results. In fact, the tall Indian jointvetch plant type has reduced the survival resources of rice, thereby seriously restricting its yield. However, research on Indian jointvetch in rice fields is limited.

Given the serious harm caused by Indian jointvetch in rice fields, genetic research is important to gain a deeper understanding of this weed and predict its evolution in rice fields in the future. Because the cp. genome has unique research value in phylogeny, species identification, and population genetics [21], we sequenced the cp. genome of Indian jointvetch for the first time in this study, demonstrating a typical tetrad structure, including genes encoding proteins, tRNAs, and rRNAs, similar to the cp. genomes reported in other plants [22, 34, 41]. The complete cp. genome of Indian jointvetch was 163,613 bp long, which is larger than that of other common plants found in rice fields with reported cp. genomes, including those of the genera *Oryza* [42], *Echinochloa* [41], and *Ammannia* [34].

Repeating sequences are unique labels of a species, including SSRs and LRs. SSRs, composed of 1–6 nucleotide repeat units, are widely distributed molecular genetic markers in the cp. genome of plants [43, 44]. We identified 161 SSRs in the cp. genome of Indian jointvetch, which can be used for population genetics analyses and plant genotyping [45–48]. We also identified 166 LRs in the Indian jointvetch cp. genome. LRs are a special type of DNA repeat sequence that typically occupy a large proportion of the genome [49]. These repetitive structures exhibit diversity in the cp. genome and promote molecular recombination [50], which has important molecular and biological significance in the study of plant evolution [51]. Thus, the SSRs and LRs detected in this study offer important biological information resources for the identification of Indian jointvetch and to facilitate genetic diversity and population structure research of *Aeschynomene*.

In order to explore the closely related plants of Indian jointvetch, we conducted phylogenetic tree analysis, IR expansion and contraction comparisons, and SNP studies based on the cp. genome. Genome assembly and characterization provides great value in defining species, revealing phylogenetic relationships, and determining taxonomic status [52–55]. Phylogenetic analysis based on the cp. genomes showed that species with a close genetic relationship to Indian jointvetch included *A. sinicus*, *M. polymorpha*, *G. soja*, *G. max*, and *Sesbania cannabina* voucher IBSC. Notably, the genetic relationship between the two species of *S. cannabina* registered in the NCBI database is not close, suggesting that they may in fact represent different species or diverged due to geographical isolation and local adaptation. IR expansion and contraction in the cp. genome are common phenomena in plants [18], mainly occurring at the IR/SC junction [56], which is a key driving force in plant evolution [57–60]. The results of IR expansion and contraction analyses further indicated that the genetic relationship between Indian jointvetch and *S. cannabina* voucher IBSC was closer. Because their boundary genes are completely identical, except for differences in length of the genes or the distance between the genes and the boundary (Fig. 4). SNPs are important indicators of evolutionary differences between closely related species. These direct molecular markers can clearly display the exact nature and location of allele variations [61]. Using the cp. genome of Indian jointvetch as reference, we identified 3281, 3840, and 3838 SNPs in the cp. genomes of *S. cannabina* voucher IBSC, *G. soja*, and *G. max*, respectively. The fewer SNPs detected represents the fewer variations in single nucleotides. This result further indicated that the Indian jointvetch and *S. cannabina* voucher IBSC have a relatively close genetic relationship, which is consistent with the phylogeny and IR expansion and contraction results. These SNPs can be used as important differential nucleotide databases to distinguish the species.

From the perspective of crop protection, it is necessary to study reasonable measures to eliminate Indian jointvetch from paddy fields. The advantages of chemical methods for weed control in upland direct-seeding rice fields have been reported for decades in terms of higher yields and lower labor costs [30–32]. However, the chemical control of Indian jointvetch is currently very challenging [3]. Our results also indicated that the availability of herbicides for Indian jointvetch control is limited. Herbicides commonly used to control broadleaf weeds in rice fields in China, such as bensulfuron-methyl and 2-methyl-4-chlorophenoxyacetic acid [62–64], had poor effects on inhibiting the germination of Indian jointvetch. Nevertheless, we found that among the 10 herbicides tested, four met the requirements, including two pre-emergence herbicides (saflufenacil and oxyfluorfen) and two post-emergence herbicides (florpyrauxifen-benzyl and penoxsulam). Given that mixing Indian jointvetch seeds with rice seriously reduces the quality of the rice [1], we strongly recommend using herbicides containing these four components to control Indian jointvetch, which is rampant in rice fields; however, further research is needed to establish effective control strategies.

It should be pointed out that the understanding of the genetic characteristics of Indian jointvetch and the prediction of the evolution of related plants into rice field weeds in this study are only based on the chloroplast genome. Further research should be based on broader morphological and whole-genome. Additionally, this study only provides suggestions for chemical control of Indian jointvetch. Further research is needed on control strategies that are more conducive to biodiversity and even the value of resource utilization of this weed.

## Conclusions

In this study, we obtained the first complete cp. genome of Indian jointvetch (*A. indica*), a common weed found in upland direct-seeding rice fields in the lower reaches of the Yangtze River in China. Overall, our study indicates that species with closer affinity to Indian jointvetch include (but are not limited to) *Glycine soja*, *Glycine max*, and *Sesbania cannabina* based on the cp. genome. Therefore, it is necessary to be vigilant about these plants becoming farmland weeds (especially *S. cannabina*), entering rice fields, and causing harm to rice. Note that because *A. sinicus* and *M. polymorpha* do not have complete IR regions, further analyses of these two species were not conducted in this study, which may warrant further exploration of their potential harm in upland direct-seeding rice fields. Toxicity evaluation of common herbicides currently used in rice fields indicate the need for the combination of herbicides to achieve effective control of Indian jointvetch and improve the yield and quality of upland direct-seeding rice.

### Electronic supplementary material

Below is the link to the electronic supplementary material.

Additional File 1

Additional File 2

Additional File 3

## Data Availability

The datasets generated and/or analyzed during the current study are available in the [National Center for Biotechnology Information] repository, [Accession number: PRJNA963187].

## Abbreviations

cp: Chloroplast
GO: Gene Ontology
GR50: The herbicide dose needed to cause 50% growth reduction of plant
IBSC: South China Botanical Garden, Chinese Academy of Sciences
IR: Inverted Repeat
KEGG: Kyoto Encyclopedia of Genes and Genomes
LR: Long repeat
LSC: Large single copy
ML: Maximum likelihood
MRCA: Most recent common ancestor
NCBI: National Center for Biotechnology Information
SSC: Small single copy
SNP: Single nucleotide polymorphism
SSR: Simple sequence repeat
