# Supplementary material for: Complete chloroplast genome and comparison of herbicides toxicity on Aeschynomene indica (Leguminosae) in upland direct-seeding paddy field
Source: BMC Genomics. 2024 Mar 14;25:277. doi: 10.1186/s12864-024-10102-x (PMC10938726; doi:10.1186/s12864-024-10102-x)
Supplement: Supplementary file 3 — Additional File 3 [file 12864_2024_10102_MOESM3_ESM.docx]

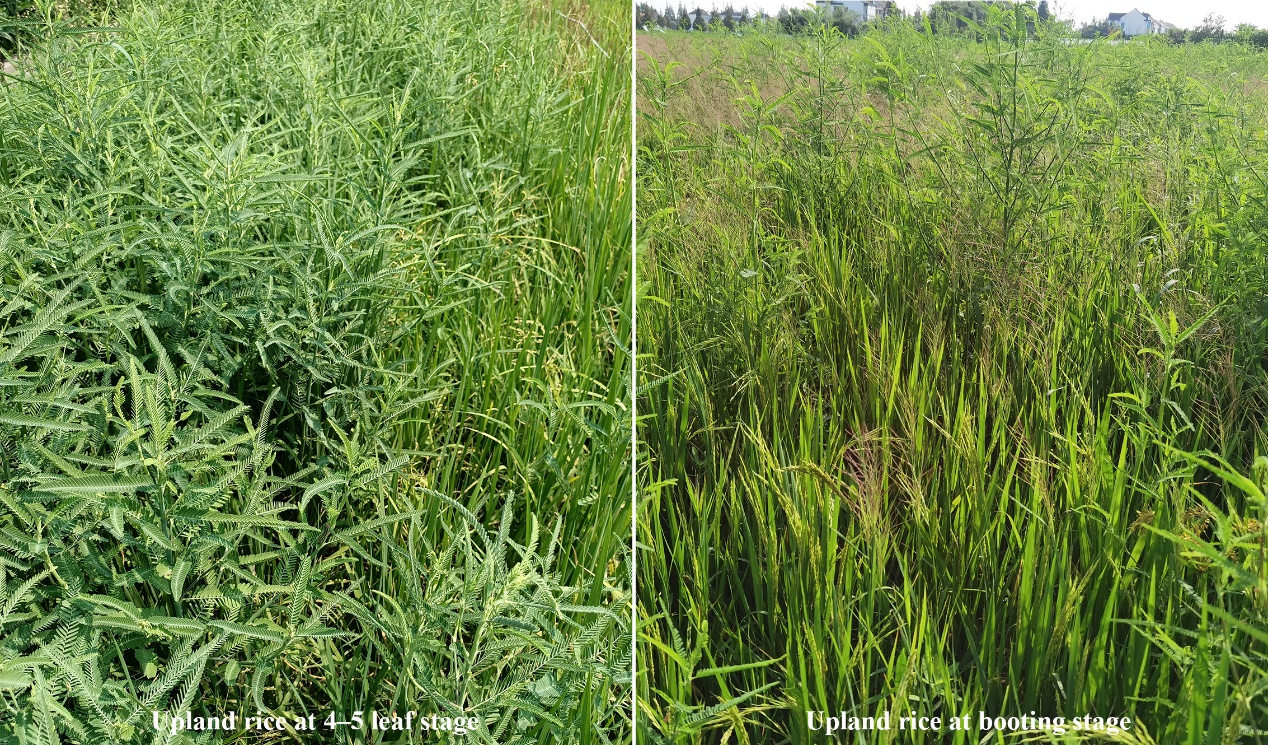
Figure S1 Damage of Indian jointvetch to upland direct-seeding rice.


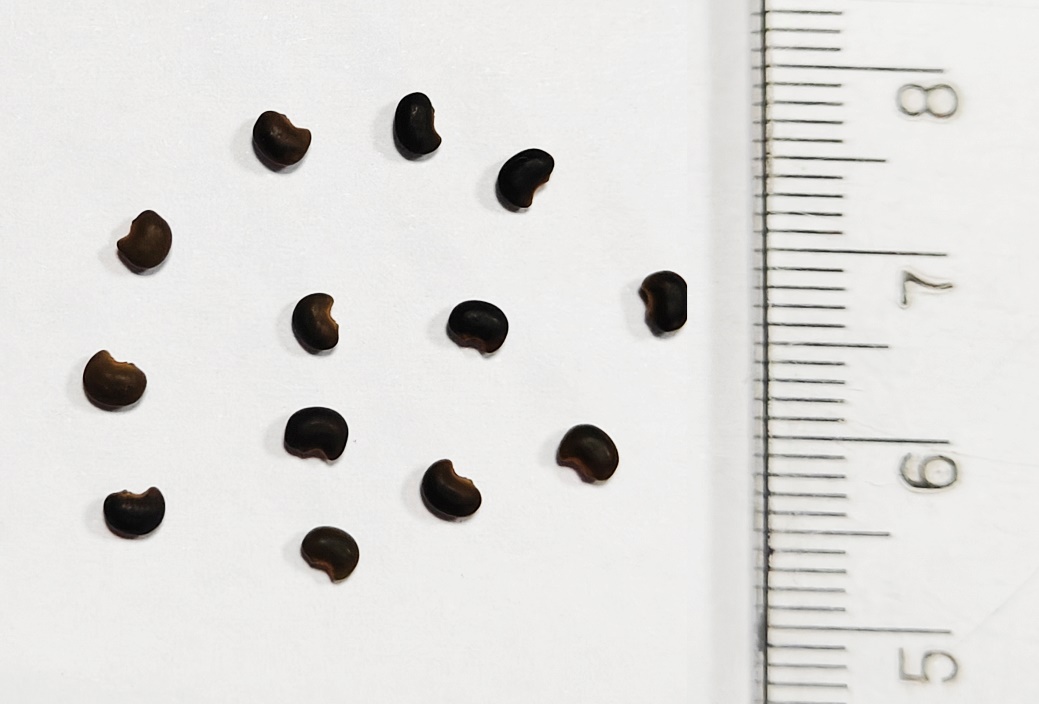


Figure S2 Morphology of seeds of Indian jointvetch. The minimum scale on the ruler represents millimeters.


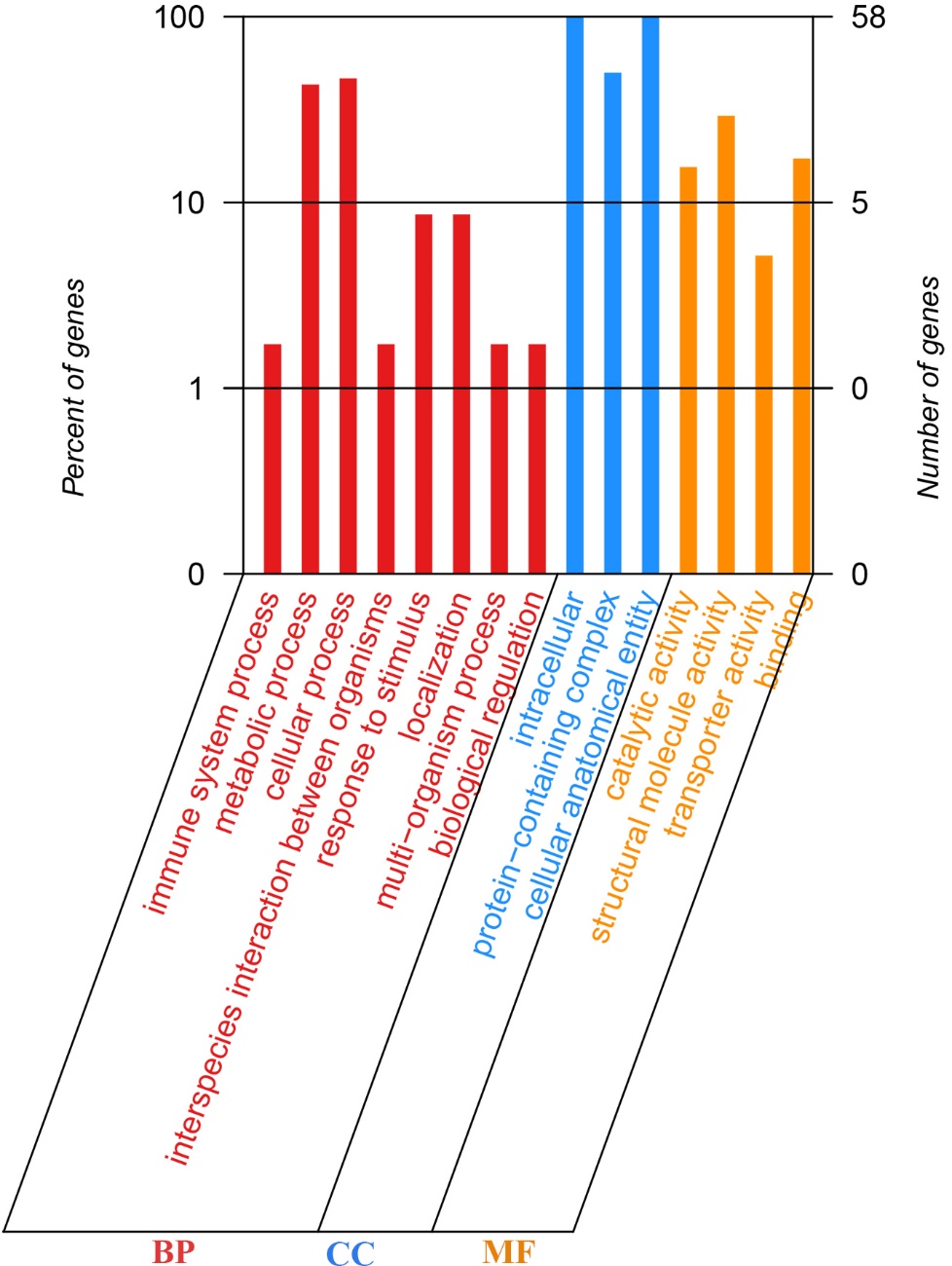


Figure S3 Percentages of Indian jointvetch chloroplast genes matched to Gene Ontology (GO) function classification. BP, biological process; CC, cellular component; MF, molecular function.


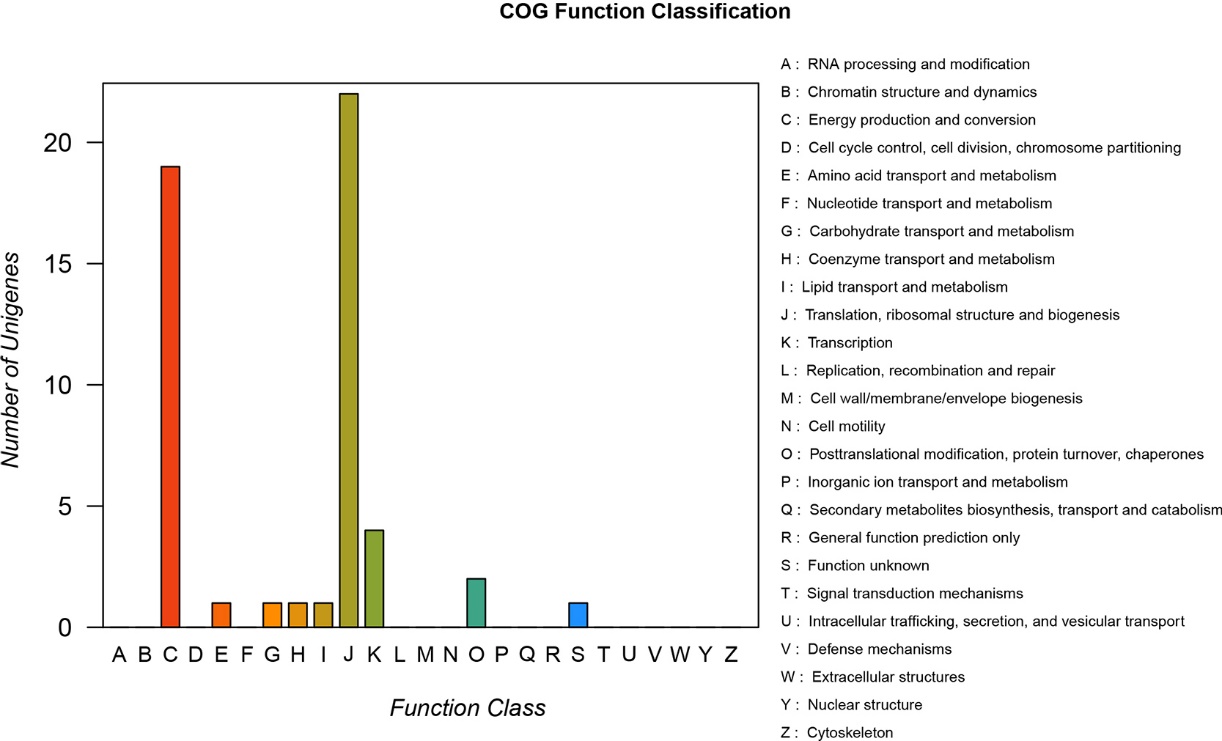


Figure S4 Number of Indian jointvetch chloroplast unigenes matched to Clusters of Orthologous Groups (COG) function classification.
